# Short-Term Changes in Metabolic Dysfunction-Associated Steatotic Liver Disease (MASLD) and Related Circulating Biomarkers Following a Very-Low Calorie Ketogenic Diet (VLCKD): A Paired Longitudinal Analysis

**DOI:** 10.3390/nu18203397

**Published:** 2026-10-16

**Authors:** Rossella Tatoli, Rossella Donghia, Pasqua Letizia Pesole, Martina Di Chito, Dolores Stabile, Annalisa Ferro, Anna Ancona, Gianluigi Casimo, Gianluigi Giannelli

**Affiliations:** 1 Data Science Unit, National Institute of Gastroenterology IRCCS “Saverio de Bellis”, 70013 Bari, Italy; rossella.tatoli@irccsdebellis.it (R.T.); gianluigi.casimo@irccsdebellis.it (G.C.); 2 Core Facility Biobank, National Institute of Gastroenterology IRCCS “Saverio de Bellis”, 70013 Bari, Italy; letizia.pesole@irccsdebellis.it (P.L.P.); dolores.stabile@irccsdebellis.it (D.S.); anna.ancona@irccsdebellis.it (A.A.); 3 Center of Nutrition for the Research and the Care of Obesity and Metabolic Diseases, National Institute of Gastroenterology IRCCS “Saverio de Bellis”, 70013 Bari, Italy; martina.dichito@irccsdebellis.it; 4 Laboratory of Clinical Pathology, National Institute of Gastroenterology IRCCS “Saverio de Bellis”, 70013 Bari, Italy; annalisa.ferro@irccsdebellis.it; 5 Scientific Direction, National Institute of Gastroenterology IRCCS “Saverio de Bellis”, 70013 Bari, Italy; gianluigi.giannelli@irccsdebellis.it

**Keywords:** MASLD, VLCKD, liver steatosis, body composition, adipose dysfunction

## Abstract

Background: Metabolic dysfunction-associated steatotic liver disease (MASLD) is the most common chronic liver disease worldwide. Its prevalence is constantly increasing, particularly among obese individuals. Lifestyle modifications remain the first-line intervention for the management and treatment of MASLD. The most widely recommended dietary regime for MASLD is the Mediterranean Diet (MedDiet). Despite its countless benefits, this dietary regimen is difficult to follow in some cases. This study aimed to assess the effects of an 8-week Very Low-Calorie Ketogenic Diet (VLCKD) in obese patients with MASLD. Methods: The study included 55 obese adults aged 18–64 years, recruited from the Center of Nutrition for Obesity and Metabolic Diseases, National Institute of Gastroenterology “Saverio de Bellis” Research Hospital (Castellana Grotte, Bari, Italy). Anthropometry, body composition (BIA), liver status (FibroScan^®^), serum biochemistry and circulating biomarkers of adipose dysfunction were assessed at baseline (T0) and post-intervention (T1). Results: Significant improvements in liver steatosis (CAP *p* < 0.0001), liver stiffness (*p* = 0.0005), anthropometric parameters (waist *p* < 0.0001), and body composition (FM *p* <0.0001) were observed. A significant reduction in leptin levels and an increase in sex hormone-binding globulin (SHBG *p* < 0.0001) were also observed. Conclusions: In this uncontrolled pre-post analysis, the 8-week VLCKD intervention was associated with improvements in hepatic steatosis, body weight, and fat mass, and selected circulating biomarkers of adipose tissue dysfunction in adults with obesity and MASLD.

## 1. Introduction

Metabolic dysfunction-associated steatotic liver disease (MASLD) is a recently defined clinical condition. This nomenclature was introduced in 2023 to replace the previous term Non-Alcoholic Fatty Liver Disease (NAFLD) [1,2,3]. This change aimed to emphasize the close relationship between metabolic syndrome and liver steatosis, which is considered a hepatic manifestation of metabolic syndrome [4].

To date, MASLD affects between 25 and 33% of the world’s population, making it the most common chronic liver disease [5]. It is defined by the presence of liver steatosis in association with one or more metabolic risk factors, such as overweight and obesity, hypertension, type 2 diabetes, fasting hyperglycemia, and elevated cholesterol, in the absence of significant alcohol consumption [2]. The MASLD marks the onset of a disease spectrum, which begins with simple liver steatosis and may subsequently progress to Metabolic dysfunction-associated steatohepatitis (MASH), fibrosis, cirrhosis, and MASH-related hepatocellular carcinoma (HCC) [6]. MASLD is associated with an increased risk of cardiovascular events, chronic kidney disease, hepatic and extrahepatic malignancies, and progressive liver-related complications, including end-stage liver disease and HCC [7].

Global mortality from MASLD has risen over the last 10 years, with significant increases in mortality rates from liver-related and cardiovascular causes, and from non-liver cancer [8]. A further increase is expected over the next 20 years, particularly among people aged ≥65 years, non-Hispanic White and Hispanic individuals, and non-metropolitan populations [8]. Thus, MASLD significantly contributes to the global burden of liver disease-related mortality [9].

The prevalence of MASLD is higher among individuals with obesity: the reported prevalence is 80% among obese, compared with 16% among with those normal weight [10]. MASLD is closely related to obesity, insulin resistance, and type 2 diabetes [11]. Obesity is characterized by a generalized inflammatory state that, through various biochemical and metabolic mechanisms, affects the liver, promoting fat deposition and the development of liver steatosis [12]. Previous evidence suggests that Visceral Adipose Tissue (VAT) plays a central role in this link, as it can produce and secrete numerous cytokines known as adipokines [13]. Adipose tissue is now fully recognised as an endocrine organ, capable of secreting biologically active peptides, known as adipokines, which exert effects on various organs, including the liver [14]. Adipokines are involved in regulating glucose and lipid metabolism, improving insulin resistance and maintaining general homeostasis. An increasing number of studies suggest an interaction between adipose tissue and the liver, which plays a key role in the onset of metabolic diseases such as MASLD [15,16]. Through the secretion of specific hormones and adipokines, adipose tissue can communicate with the liver, influencing the function of liver cells [17]. For example, the FGF21-adiponectin axis acts as a key mediator of the interaction between the liver and adipose tissue, playing a role in maintaining energy expenditure homeostasis [15,16]. Chemerin has anti-inflammatory and antioxidant effects, reducing oxidative stress and levels of inflammatory proteins [18,19]. Vifastin, on the other hand, has pro-inflammatory effects, increasing lipid accumulation and stress in the endoplasmic reticulum [20,21]. Within the liver, leptin acts by effectively reducing the accumulation of lipids whilst promoting their mobilization [22]. Maintaining physiological levels of leptin is essential for preventing hepatic steatosis. In MASLD, leptin can take on pro-inflammatory and pro-fibrotic functions [23]. Visfatin influences various metabolic pathways, including the response to oxidative stress, cellular apoptosis, lipid and glucose metabolism, insulin resistance and inflammation. Its involvement in these pathways potentially contributes to the pathogenesis of MASLD [24].

Although there is increasing discussion of pharmacological therapies aimed at treating MASLD [25], the first-line approach remains non-pharmacological and is based on lifestyle changes [26]. It has been shown that even a 5% reduction in body weight, achieved through adopting a healthy diet and an active lifestyle, has a positive effect on reducing liver fat accumulation, improving insulin sensitivity and glycemic profile in patients with MASLD, both with and without diabetes [27,28]. The combination of dietary intervention and physical activity appears to have greater potential benefits than either intervention implemented separately [29]. Caloric restriction leads to a rapid improvement in fatty liver disease and hepatic insulin resistance, as well as greater weight loss, with all resulting positive metabolic effects [30]. In fact, a weight loss of 10% or more appears to lead to a complete resolution of fatty liver disease [28].

The Mediterranean Diet (MedDiet) is recommended for managing MASLD [31]. Owing to the great variety of specific antioxidants, the MedDiet helps counteract the oxidative stress and inflammation associated with steatosis [32]. The benefits of the MedDiet for patients with MASLD can also be attributed to the presence, in the foods that characterise it, of monounsaturated fatty acids, dietary fibre and proteins, which are of high biological value and easily digestible [33]. Despite its proven benefits, the MedDiet is not without its potential drawbacks. Economic factors, such as the cost of Extra Virgin Olive Oil (EVOO), fresh products, and fish, may affect affordability and make it difficult to stick to this diet [34]. It is always important to keep an eye on your calorie intake. Whilst generally healthy, even the MedDiet can be high in calories if you consume excessive amounts of its characteristic foods, leading to weight gain [35].

Furthermore, MedDiet may be challenging to follow for populations outside the Mediterranean area owing to different socio-cultural habits [36].

The Very Low-Calorie Ketogenic Diet (VLCKD) may be considered a potentially effective alternative for weight loss and management of patients with MASLD [37]. This is a low-calorie diet, providing less than 800 kcal/day, with normal protein intake, low carbohydrate content, and high fat content [38]. VLCKD involves shifting the primary energy source from carbohydrates to fats. Consuming fewer than 50 g of carbohydrates per day causes fat to become the primary metabolic substrate, resulting in the production of ketones in the liver [38]. Initially used to treat obesity, it appears to have shown promising results in the management of MASLD as well [39]. Currently, the scientific literature does not provide sufficient evidence to determine with certainty which dietary model is most effective in the management of MASLD and its progression to more advanced stages of the pathological spectrum to which it belongs [40].

The aim of this study was to contribute to this field by assessing the effects of VLCKD on the hepatic and metabolic profiles of obese patients with MASLD.

## 2. Materials and Methods

### 2.1. Study Design and Population

This retrospective secondary analysis used prospectively collected data and biological samples from participants enrolled in the registered clinical study NCT05477212. Participant enrollment, 8-week VLCKD intervention, and collection of clinical, anthropometric, biochemical, instrumental, and biological data at baseline (T0) and post-intervention (T1) were prospectively planned and performed according to the study protocol. Subsequently, this analysis was conducted in participants with obesity and MASLD for whom the paired T0–T1 data and biological samples required for the current investigation were available. The study was conducted at the Center of Nutrition for Obesity and Metabolic Diseases, National Institute of Gastroenterology “Saverio de Bellis” Research Hospital (Castellana Grotte, Bari, Italy). For the present analysis, 55 adults with obesity (BMI ≥ 30 kg/m^2^) aged between 18 and 64 years were recruited. According to the exclusion criteria established for the study, participants with type 1 diabetes mellitus, severe cardiovascular or respiratory diseases, gastrointestinal disorders, chronic kidney disease, psychiatric illnesses, pregnancy or lactation, eating disorders, substance abuse, active infections, or rare metabolic or genetic conditions were ineligible. Excessive alcohol consumption (>20 g/day for women and >30 g/day for men) was an exclusion criterion. Information on smoking habits and alcohol consumption was collected using standardized interviews, as required by the American and European clinical guidelines. The study protocol was approved by the Ethics Committee of the IRCCS Oncological Hospital “Giovanni Paolo II”, Bari, Italy (approval no. 179/C.E. de Bellis, 13 May 2022) in compliance with the Declaration of Helsinki, and all participants provided informed consent.

Participant enrollment for the parent registered study began in July 2022 and ended in December 2023. All participants included in the present analysis had baseline (T0) and post-intervention (T1) assessments, with T1 performed after 8 weeks of VLCKD. The diet provided a calorie intake of 650–800 kcal/day, with a moderate protein intake and restricted carbohydrate intake (<30 g/day). The main source of fat was EVOO. Micronutrient supplementation was provided to prevent deficiencies. Patients were advised to drink at least 2 L of water daily to ensure adequate hydration. Dietary adherence was monitored by daily food diaries.

### 2.2. Anthropometric Parameters

Anthropometric parameters were measured using calibrated instruments at both T0 and T1. All patients were measured using the same calibrated scale and stadiometer. The patients removed their outer garments and stood with their feet close together for waist circumference measurement. The circumference point was situated at the lower rib margin and iliac crest. To calculate BMI (kg/m2), the weight and height of all patients were recorded in the same manner, using the same set of scales and stadiometer. The participants were barefoot, fasting, wearing light clothing, and had empty bladders.

### 2.3. Bioelectrical Impedance Analysis (BIA)

Bioelectrical impedance analysis (BIA) was performed using a single-frequency bioimpedance analyzer (BIA-101 analyzer, 50-kHz frequency; Akern Bioresearch, Florence, Italy). The measurements were performed by specialist dietitians using the same pre-established protocol and in accordance with the guidelines set forth by the European Society of Parenteral and Enteral Nutrition (ESPEN) [41]. The Akern software (Bodygram^®^ Dashboard) was used to calculate body composition parameters.

### 2.4. Diet Protocol

The diet was drawn up by dietitians following a nutritional consultation, during which the patient’s habits and lifestyle were assessed. Furthermore, the diet was devised taking into account anthropometric parameters and daily energy expenditure as determined by BIA. The total carbohydrate intake ranged from 30–50 g/die, whilst the fat intake ranged from 20–30 g/die, predominantly of plant origin, derived from nuts and extra virgin olive oil. The protein intake ranged from 1.2–1.4 g per kg of ideal body weight per day. The consumption of specific types of vegetables was permitted without restriction. The daily calorie intake was under 800. Drinking a minimum of 2 L of water per day was advised. According to current recommendations, micronutrient supplements were prescribed during the whole dietary intervention to prevent nutritional deficits [42].

### 2.5. Liver Steatosis and Fibrosis Assessment by FibroScan^®^

Liver status, including liver stiffness and steatosis, was assessed via transient elastography (FibroScan^®^ Expert 630, Echosen Italia SRL, Verona, Italy) after a minimum six-hour fast. All measurements were performed by trained operators. Liver stiffness (kPa) and Controlled Attenuation Parameter (CAP; dB/m) were measured from the right hepatic lobe. The data quality requirements included acquiring ≥10 valid measurements and an interquartile range of less than 30% of the median to ensure reliability.

For the present analysis, MASLD eligibility was established at baseline by documenting hepatic steatosis together with at least one cardiometabolic risk factor. Hepatic steatosis was assessed by transient elastography using the Controlled Attenuation Parameter (CAP). All 55 participants included in the present analysis fulfilled the MASLD criteria at baseline [2]; obesity alone was not considered sufficient for inclusion. Alcohol exposure was assessed by standardized interview, and participants reporting >20 g/day for women or >30 g/day for men were excluded, as described above.

### 2.6. Biochemical Analyses and Hepatokines Determination

Blood samples were collected between 8:00 a.m. and 9:00 a.m. after overnight fasting. Glucose, insulin, creatinine, total protein, sodium, potassium, calcium, phosphorus, iron, uric acid, ferritin, TSH, FT3, FT4, PTH, triglycerides, total cholesterol, LDL-cholesterol, HDL-cholesterol, AST, ALT, gamma-GT, CRP, anti-TPO, NT-proBNP, testosterone, prolactin, vitamin D, concentrations were measured by a COBAS 8000 autoanalyzer (ROCHE Diagnostic SPA, Monza, Italy). HbA1c was determined using an automatic capillary electrophoresis system (Capillarys 3 OCTA; Sebia Italia S.r.l., Bagno a Ripoli, Firenze, Italy). Blood cell count was evaluated by fluorescence flow cytometry using the automatic hematology analyzer Sysmex XT-1000 (Dasit, Cornaredo, Milano, Italy). The glomerular filtration rate was estimated using the CKD-EPI formula. The determination of specific hepatokines was quantified using enzyme-linked immunosorbent assay (ELISA) on the serum of patients, following the manufacturer’s protocols. Insulin resistance was estimated using the Homeostatic Model Assessment for Insulin Resistance (HOMA-IR), calculated as insulin (μUI/mL) × glucose (mg/dL)/405.

Follistatin, resistin, SHBG, chemerin, PAI-1, visfatin, FGF-21, RBP4, leptin and adiponectin serum level were determined by Human ELISA kit, Invitrogen, Vienna, Austria. Supplementary Table S2 lists the proteins assayed and assay sensitivities.

Serum autotaxin levels were quantified using an ELISA, following the manufacturer’s protocol (Human ENPP-2/Autotaxin Immunoassay ELISA kit, R&D SYSTEM, Bio-techne, Minneapolis, MN, USA). The minimum detectable concentration of human ENPP-2 ranged between 0.055 and 0.157 ng/mL.

Lysophosphatidic acid (LPA) levels in serum were determined using an ELISA kit in accordance with the manufacturer’s instructions (Human Lysophosphatidic Acid ELISA kit, Cusabio, Houston, TX, USA). The assay sensitivity was <3.9 ng/mL.

### 2.7. Statistical Analysis

Patient characteristics are reported as median and interquartile range (IQR) for continuous variables. The Wilcoxon matched-pairs signed-rank test was applied for continuous parameters to evaluate variations after the VLCKD observation.

Missing values in circulating biomarker measurements were handled using multiple imputation by chained equations (MICE) under the assumption that the data were missing at random (MAR). Biomarkers included in the imputation procedure were follistatin, resistin, sex hormone-binding globulin (SHBG), chemerin, plasminogen activator inhibitor-1 (PAI-1), visfatin, fibroblast growth factor-21 (FGF-21), retinol-binding protein-4 (RBP4), leptin, adiponectin, lysophosphatidic acid (LPA), and autotaxin (ATX). Complete variables, including the controlled attenuation parameter (CAP), liver stiffness (FibroScan E), age, sex, and study time point (baseline/post-intervention), were included as predictors in the imputation models but were not imputed.

Multiple imputation was performed using chained equations with predictive mean matching (PMM) and five nearest neighbors (k = 5) to preserve the observed distribution of continuous variables and avoid implausible imputed values. A total of 50 imputed datasets were generated after 20 burn-in iterations using a fixed random seed to ensure reproducibility. The first completed dataset was subsequently extracted for post-imputation diagnostic assessments.

Changes in hepatic and metabolic biomarkers from baseline to post-intervention were assessed using the paired Wilcoxon signed-rank test. For each biomarker, the individual change was calculated as the post-intervention value minus the corresponding baseline value. The magnitude and direction of the within-subject change were quantified using Cohen’s d, which was calculated as the mean of the paired differences divided by their standard deviation. Negative values indicated a reduction after the VLCKD, whereas positive values indicated an increase.

To account for multiple comparisons, nominal *p*-values were adjusted using the Benjamini–Hochberg procedure to control the false discovery rate (FDR). Biomarkers were classified as significantly decreased or increased when the FDR-adjusted *p*-value was <0.05 and Cohen’s d was negative or positive, respectively. Associations with a nominal *p*-value < 0.05 but an FDR-adjusted *p*-value ≥ 0.05 were considered nominally significant.

The volcano plot displays Cohen’s d on the x-axis and −log_10_ of the nominal *p*-value on the y-axis. The horizontal dashed line corresponds to a nominal significance threshold of *p* = 0.05, while the vertical dotted lines at Cohen’s d >=−0.5 and 0.5 indicate moderate standardized effects. The point size was proportional to the absolute value of Cohen’s d, and the point color represented the direction and FDR-based statistical significance of each change.

Exploratory Spearman rank correlation analyses were performed to investigate the relationships between CAP, as a measure of hepatic steatosis, and circulating biomarkers related to adipose tissue dysfunction and metabolic regulation. Correlations were examined at baseline and post-intervention. In addition, correlations between changes (Δ) in CAP and concomitant changes (Δ) in circulating biomarkers were explored to assess whether reductions in hepatic steatosis tended to occur alongside changes in these biomarkers. Given the sample size and number of comparisons, these analyses were considered exploratory and hypothesis-generating. To test the null hypothesis of non-association, the two-tailed probability level was set at 0.05. The analyses were conducted using StataCorp. 2025. *Stata Statistical Software: Release 19*. College Station, TX: StataCorp LLC., and RStudio (version 2026.08.2, “Yellow Yarrow”; Posit Software, PBC, Boston, MA, USA).

## 3. Results

The demographic and lifestyle characteristics of the study population are reported in Supplementary Table S1. The study included 55 participants with a median age of 43.16 years (IQR: 18.32), of whom 43.64% were male. Most participants had completed at least a high school education, with 48.72% holding a high school diploma, 28.21% a university degree, and 5.13% a postgraduate qualification. Meanwhile, 17.95% had completed middle school education. Regarding marital status, 59.52% of the participants were married or cohabiting, and 40.48% were single. No participant was divorced, separated, or widowed. Regarding smoking habits, half of the study population were non-smokers (50.00%), 16.67% were former smokers, and 33.33% were current smokers.

Table 1 summarizes the changes in clinical, anthropometric, body composition, and biochemical parameters after the VLCKD intervention.

A significant reduction in both systolic and diastolic blood pressure was observed after the intervention (both *p* < 0.0001). Anthropometric measurements showed marked improvements, including reductions in body weight, body mass index (BMI), waist circumference, and neck circumference (all *p* ≤ 0.002).

Body composition analysis using bioelectrical impedance showed a significant decrease in fat mass (FM), fat-free mass (FFM), body cell mass (BCM), total body water (TBW), and extracellular water (ECW), along with an increase in electrical resistance (RZ). No significant changes were detected in reactance (XC), phase angle (PhA), or basal metabolic rate (BMR), whereas BCMI showed a borderline reduction (*p* = 0.05).

Regarding metabolic parameters, fasting glucose, glycated hemoglobin (HbA1c), fasting insulin, HOMA, total cholesterol, LDL cholesterol, HDL cholesterol, and triglyceride concentrations significantly decreased following the VLCKD (all *p* < 0.01). Serum phosphorus and sodium levels increased significantly, whereas serum iron levels decreased. Creatinine levels showed a small but significant increase, whereas the estimated glomerular filtration rate (eGFR) remained clinically stable despite a statistically significant difference. No significant changes were observed in the total protein, potassium, calcium, or albumin levels.

Thyroid function was characterized by a significant reduction in FT3 levels and a concomitant increase in FT4 concentrations, whereas the TSH level remained unchanged. Anti-thyroid peroxidase antibody (anti-TPO) levels were borderline increased (*p* = 0.05). Testosterone concentrations increased significantly after the intervention, while prolactin, vitamin D, intact parathyroid hormone, and NT-proBNP did not significantly change.

Hematological analysis revealed modest but significant increases in red blood cell count and hemoglobin levels, whereas platelet count, white blood cell count, absolute lymphocyte count, and absolute basophil count significantly decreased. No significant differences were found for hematocrit, erythrocyte indices (MCV, MCH, MCHC, RDW-CV), neutrophils, monocytes, eosinophils, or most leukocyte percentages.

Serum protein electrophoresis showed minor but statistically significant reductions in the α2 and β1 fractions expressed as percentages, as well as in the α2 fraction expressed as g/dL, whereas the remaining electrophoretic fractions and albumin-to-globulin ratio did not significantly change.

Furthermore, the same analysis was conducted stratified by sex (Table S3).

The changes in liver-related biochemical and instrumental parameters are summarized in Table 2, and relative stratification by sex (Table S4).

Following the VLCKD intervention, serum uric acid levels increased significantly (*p* = 0.0001), whereas gamma-glutamyl transferase (GGT) levels showed a marked reduction (*p* < 0.0001). A small but statistically significant change in C-reactive protein (CRP) was also observed (*p* = 0.04), although the median values remained within the normal range. No significant changes were detected in the aspartate aminotransferase (AST), alanine aminotransferase (ALT), or ferritin concentrations.

FibroScan assessment demonstrated a significant improvement in liver status after the intervention. Specifically, the controlled attenuation parameter (CAP), an indicator of hepatic steatosis, was markedly reduced (*p* < 0.0001), whereas liver stiffness (E), also showed a significant decrease (*p* = 0.0005). Overall, these findings indicate a substantial improvement in hepatic steatosis, along with a modest but significant reduction in liver stiffness following the VLCKD.

Table 3 summarizes the changes in circulating biomarkers following the VLCKD intervention. A marked increase in sex hormone-binding globulin (SHBG) was observed after the intervention (*p* < 0.0001).

Conversely, significant reductions were detected in several biomarkers associated with adipose tissue dysfunction, inflammation, and metabolic regulation, including chemerin (*p* = 0.005), plasminogen activator inhibitor-1 (PAI-1) (*p* = 0.01), fibroblast growth factor 21 (FGF-21) (*p* = 0.02), retinol-binding protein 4 (RBP4) (*p* = 0.006), and leptin (*p* < 0.0001). Among these, leptin exhibited the largest reduction, indicating a substantial improvement in adipose tissue endocrine activity following the VLCKD.

No significant changes were observed in the circulating follistatin, resistin, visfatin, adiponectin, lysophosphatidic acid (LPA), or autotaxin (ATX) concentrations (all *p* > 0.05).

Overall, the biomarker profile was characterized by a significant decrease in several mediators involved in adipose tissue dysfunction, insulin resistance, and hepatic metabolism, accompanied by a marked increase in SHBG, suggesting an overall improvement in metabolic homeostasis after VLCKD intervention.

With the same approach, biomarkers were analyzed in each sex (Table S5).

Figure 1 illustrates the paired changes in hepatic and metabolic biomarkers following the ketogenic dietary intervention, displaying both the standardized paired effect size (Cohen’s d) and statistical significance of the observed changes.

The most pronounced reductions were observed for the controlled attenuation parameter (CAP) and leptin, which exhibited the largest effect sizes (both Cohen’s d< −1.4) and the strongest statistical significance (both *p* < 0.0001), indicating substantial improvements in hepatic steatosis and adipose tissue-related endocrine activity, as shown in Figure 1.

Significant decreases were also detected for retinol-binding protein 4 (RBP4), plasminogen activator inhibitor-1 (PAI-1), chemerin, and fibroblast growth factor-21 (FGF-21), although with smaller effect sizes ranging from small to moderate values.

Among the biomarkers showing significant increases, sex hormone-binding globulin (SHBG) demonstrated the largest positive effect size (Cohen’s d >≈ 0.55) and the strongest statistical significance, indicating a marked change in this circulating biomarker following the intervention.

Several additional biomarkers, including adiponectin, visfatin, lysophosphatidic acid (LPA), autotaxin (ATX), follistatin, and resistin, did not show statistically significant changes after correction for multiple testing, despite nominal trends.

Overall, the pattern of changes indicates that VLCKD was associated with marked improvements in biomarkers related to hepatic steatosis, adipose tissue dysfunction, and insulin resistance, with the largest effects observed for CAP, leptin, and SHBG.

Figure 2 shows the Spearman correlation matrices among the hepatic and metabolic biomarkers under the three study conditions (panels A–C).

Overall, the correlation structure remained relatively stable across the three analyses, with most pairwise associations being weak to moderate. Both positive and negative correlations were observed, although most did not reach statistical significance.

Across all three panels, the strongest inverse correlations consistently involved leptin, which showed moderate-to-strong negative associations with several biomarkers, particularly with RBP4 and FGF-21. Conversely, moderate positive correlations were observed among biomarkers related to adipose tissue dysfunction and metabolic regulation, including adiponectin, chemerin, PAI-1, and visfatin, although the strength and statistical significance of these associations varied across different analyses.

CAP exhibited predominantly weak correlations with circulating biomarkers, with only a few nominally significant associations and no evidence of strong relationships. Similarly, SHBG, LPA, and ATX displayed generally weak correlation coefficients, suggesting a limited association with the remaining biomarkers.

Comparison of panels A, B, and C revealed only modest changes in the overall correlation network after the intervention. Although some individual correlations became stronger or weaker, exploratory correlation analyses showed predominantly weak-to-moderate associations between CAP and circulating biomarkers at baseline and post-intervention. Similarly, correlations between changes in CAP and concomitant changes in biomarkers were generally weak, with only a limited number of significant associations. Given the exploratory nature of these analyses and the number of comparisons performed, these findings should be interpreted with caution.

## 4. Discussion

This study assessed the metabolic and hepatic effects of 8 weeks of VLCKD in obese patients with MASLD.

Several studies have analyzed the potential impact of different dietary patterns on the management of metabolic disorders. Although the MedDiet remains the most widely recommended model, its practical application presents potential challenges of a social, cultural, and economic nature, particularly outside the Mediterranean region. Whether VLCKD represents an alternative dietary strategy to the MedDiet in patients with MASLD cannot be established from the present study and requires direct evaluation in randomized comparative trials.

Our results showed in the study sample a significant improvement in the parameters associated with MASLD after VLCKD. Positive effects were observed on liver steatosis, weight loss, anthropometric measurements, body composition, and metabolic biomarkers.

In line with previous studies [43,44], we identified a significant reduction in CAP, a parameter indicating the accumulation of liver fat as measured by Fibroscan, following treatment with VLCKD. This liver-protective effect may be attributable to carbohydrate and calorie restriction, which triggers ketogenesis [45]. The reduction in liver fat accumulation can also be a direct consequence of decreased insulin resistance, which is one of the cornerstones of MASLD pathogenesis [46,47,48]. The observed reduction in CAP occurred in parallel with substantial weight loss and marked dietary energy and carbohydrate restriction. Given the uncontrolled design, the present study cannot determine the relative contribution of weight loss, caloric restriction, carbohydrate restriction, ketosis, or other components of the VLCKD to the observed improvement in hepatic steatosis. Insulin resistance remains a central component of MASLD pathogenesis [46,47,48]. In addition to the reduction in CAP, our results also showed a reduction in E, another parameter measured using Fibroscan. A decrease in this indicator post-VLCKD suggests that this dietary pattern could also be used in MASH, the next stage of MASLD. However, this hypothesis requires further investigation.

From a blood chemistry perspective, we observed an increase in uric acid levels and a decrease in iron levels. The rise in uric acid is a consequence of the ketosis itself. During a ketogenic state, with the accumulation of ketone bodies, the constant intracellular anion gradient forces the urate transporter 1 (URAT1) to reabsorb urate into the cells [49]. Previous studies have shown that the activity of a Na^+^-monocarboxylate anion co-transporter, which recycles these anions from the apical membrane of the tubular lumen into the peritubular cells, can be inhibited by short-chain fatty acids [50,51]. When fatty acids are metabolised in the liver during prolonged calorie restriction on a ketogenic diet, their inhibitory effect diminishes. However, in most cases this is a transient condition. It appears that a reduction in iron levels should also be regarded as a potential direct effect of nutritional ketosis induced by the VLCKD [52].

The change in thyroid hormone levels is an interesting finding, which has also been observed in previous studies involving the VLCKD. The decrease in FT3 levels and the increase in FT4 following the VLCKD appear to suggest that this dietary regimen induces a reduction in iodothyronine deiodase, thereby reducing the production of free T3 and circulating hormone levels [44].

After treatment with VLCKD, improvements were also observed in anthropometric parameters (BMI, waist, neck) and in the percentage of body Fat Mass (FM) measured using BIA. A minimal reduction in body Free Fat Mass (FFM) was also observed, in line with that recorded for other dietary protocols used in the management of MASLD [53]. This reduction is also in line with other studies in which the VLCKD was used for patients with MASLD [54].

No direct measurements of Visceral Adipose Tissue (VAT) were available. The observed reduction in waist circumference may suggest an improvement in abdominal adiposity; however, as waist circumference is only a surrogate marker, no direct conclusions regarding changes in VAT can be drawn. [55]. VAT appears to play a different role than subcutaneous adipose tissue in the development and progression of MASLD. It is characterized by greater lipolytic activity, greater insulin resistance, and increased secretion of pro-inflammatory and pro-fibrogenic molecules [17,56]. Therefore, a reduction in VAT leads to an improvement in MASLD through a reduction in fat deposits, both locally and in the liver, increased insulin sensitivity, and a reduction in the level of systemic inflammation.

Adipose tissue dysfunction can be regarded as the initial stage in the pathogenesis of MASLD [25]. Overeating and a chronic positive energy balance lead to excessive accumulation of lipids in White Adipose Tissue (WAT). In an attempt to compensate for excess lipids, adipose tissue undergoes hypertrophy and hyperplasia, resulting in tissue hypoxia, inflammation, and insulin resistance [57]. The latter triggers lipolysis in adipose tissue, leading to the release of Non-Esterified Fatty Acids (NEFA) into the bloodstream, which are then stored in hepatocytes as triglycerides. In the liver, the oxidation of NEFAs generates oxidative stress, inflammation, and hepatic insulin resistance [58]. Insulin resistance, which persists in both the liver and WAT, causes impaired inter-organ communication, normally mediated by adipokines, hepatokines, and other hormones [59]. The improvement in insulin sensitivity in MASLD following treatment with VLCKD consequently leads to improvements in several fronts. Our results showed significant reductions in several metabolic parameters, including blood glucose, glycated hemoglobin, total cholesterol, LDL cholesterol, triglycerides, and GGT, whereas AST and ALT showed no statistically significant changes. We also observed a reduction in circulating leptin levels and an increase in sex hormone-binding globulin (SHBG) levels.

In severe obesity, elevated leptin levels are observed due to the overexpression of the leptin gene in adipose tissue [60], and due to an impairment of leptin’s effects attributable to a condition known as ‘leptin resistance’ [61]. In line with scientific literature, our results showed a reduction in leptin levels following the VLCKD. Balestra et al. demonstrated a marked decrease in the leptin-to-adiponectin ratio, indicating an improvement in the endocrine-inflammatory balance of adipose tissue [62].

Insulin resistance, typically observed in obesity and MASLD, is associated with reduced levels of SHBG [63]. Some studies suggest that the increase in SHBG levels following dietary intervention may not be attributable to an improvement in the lipid and glucose profiles or weight loss, but rather to an improvement in liver steatosis [64]. Telgenkamp et al. estimated that approximately 20% of the increase in serum SHBG levels is mediated by a reduction in intrahepatic fat [65]. This finding, in line with our results, leads us to hypothesize that the increase in SHBG following VLCKD may reflect an improvement in hepatic metabolism. However, further studies are required to support this hypothesis.

### Study Limitations

Although the present study provides novel insights into the effects of VLCKD on MASLD and adipose tissue dysfunction, some limitations should be acknowledged in this study. The relatively short duration of the study did not allow for the assessment of the long-term maintenance of metabolic benefits. Future studies with longer follow-up periods and additional diagnostic techniques may further clarify the mechanisms underlying the observed improvements and confirm the durability of these preliminary findings. Furthermore, liver stiffness was not assessed histologically. Therefore, the observed change in transient-elastography-derived liver stiffness cannot be interpreted as evidence of liver stiffness regression, particularly considering the short duration of the intervention and the potential influence of changes in steatosis, inflammation, and metabolic status on liver stiffness measurements. The small sample size limits the generalizability of the results. A major limitation is the absence of a control group. Consequently, the pre-post design does not allow causal attribution of the observed changes specifically to VLCKD, nor does it permit differentiation between the effects of weight loss, caloric restriction, carbohydrate restriction, ketosis, and other components of the intervention. Moreover, because no Mediterranean diet comparator was included, no conclusions regarding the comparative efficacy of VLCKD and the Mediterranean diet can be drawn. Randomized controlled trials including weight- and/or energy-matched dietary comparators are needed to address these questions. Another limitation is the lack of data on body composition collected using Dual-Energy X-ray Absorptiometry (DEXA). It provides more accurate information on body composition and distinguishes between visceral and subcutaneous adipose tissues, data that cannot be obtained through other assessments. Our study provides information on body composition assessed using anthropometric measurements and Bioelectrical Impedance Analysis (BIA). This choice was based on the greater practicality and lower invasiveness of these measurements, particularly given the short time interval between T0 and T1.

## 5. Conclusions

Lifestyle changes remain the primary intervention for treating MASLD. In this uncontrolled pre-post study, the 8-week VLCKD intervention was associated with improvements in body weight, hepatic steatosis, body fat, and selected circulating biomarkers of metabolic dysfunction in adults with obesity and MASLD. Because no control or Mediterranean diet comparator was included, these findings do not establish a VLCKD-specific effect or comparative efficacy versus the MedDiet. Whether VLCKD may represent an alternative dietary strategy for selected patients with MASLD should be evaluated in adequately powered randomized controlled trials with appropriate dietary comparators.

However, we believe that a few clarifications are necessary. VLCKD is not nutritionally balanced, characterized by a high fat content and a low carbohydrate intake, which could cause a spike in plasma levels of total cholesterol and triglycerides. Particular attention should also be paid to the choice of fats consumed, favoring unsaturated fats over saturated fats, for example, EVOO and nuts. Restricted intake of fruits and vegetables may contribute to the onset of certain side effects, such as constipation [53]. Other potential side effects include transient hypoglycaemia, halitosis, hyperuricaemia and gastrointestinal disturbances, which can be effectively managed through well-structured protocols and appropriate clinical and laboratory monitoring.

Therefore, it is advisable to adopt VLCKD under close supervision of a doctor or healthcare professional [53].

## Figures and Tables

**Figure 1:**
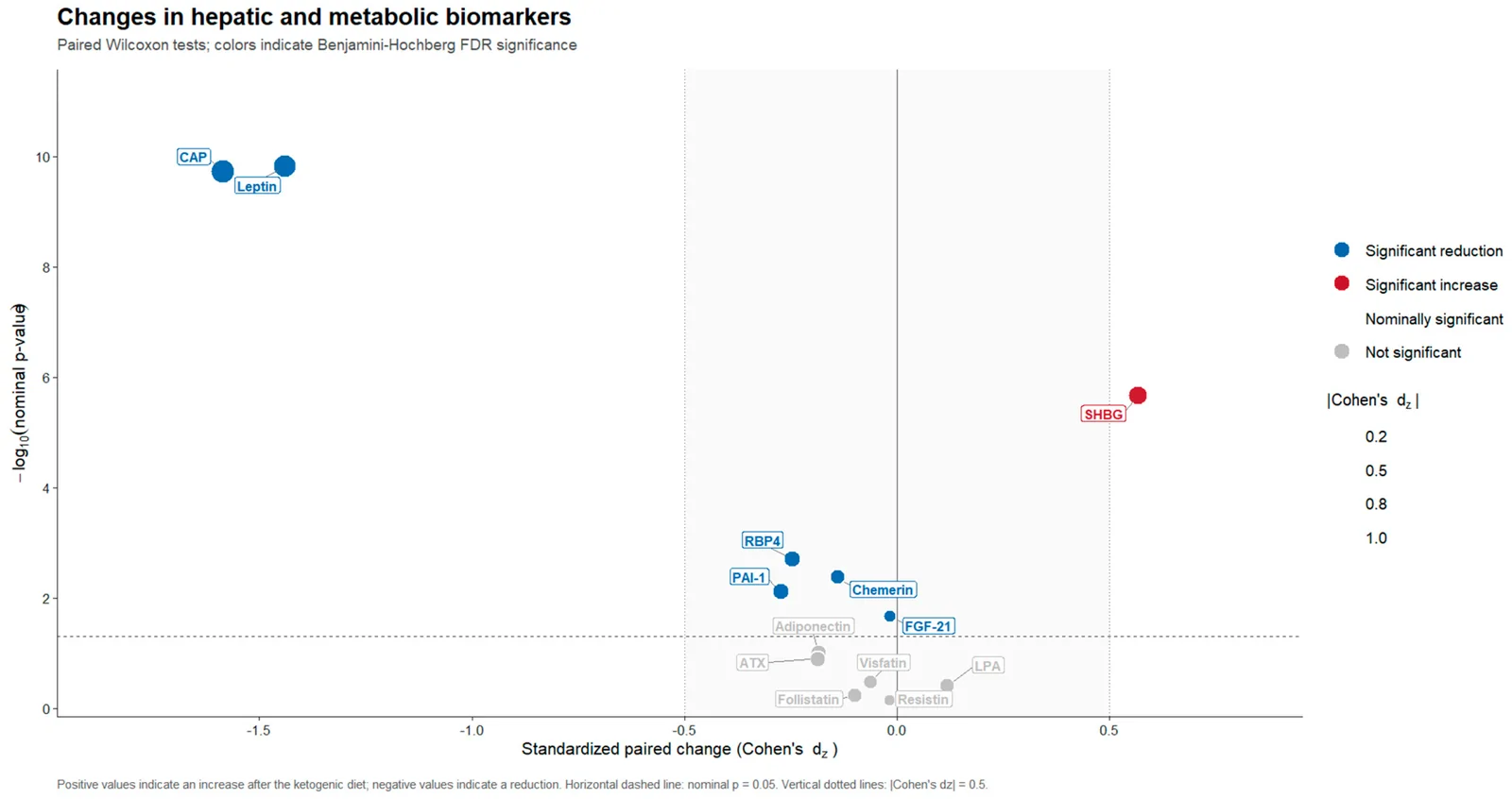
Volcano plot showing changes in hepatic and metabolic biomarkers following dietary intervention. The x-axis represents the standardized paired change, and the y-axis represents the nominal significance. Colors indicate significance after Benjamini–Hochberg FDR correction (blue: significant decrease; red: significant increase; orange: nominally significant; gray: not significant). The point size is proportional to the absolute effect size.

**Figure 2:**
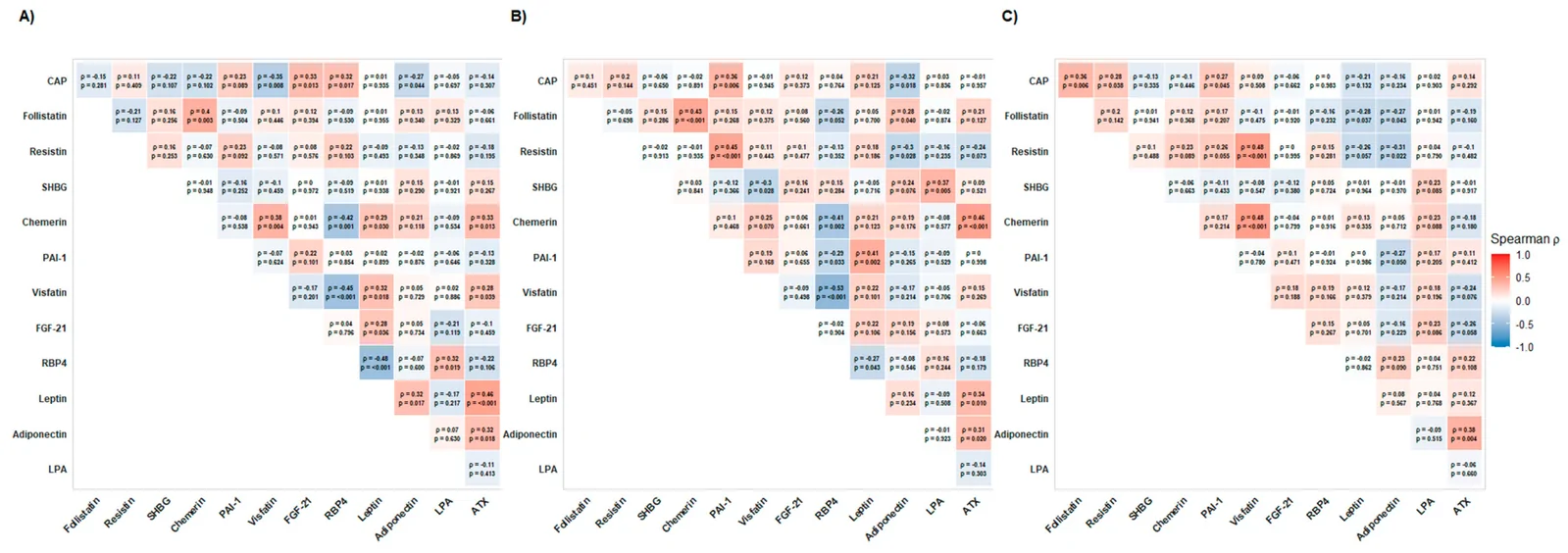
Spearman correlation heatmaps showing the associations between the Controlled Attenuation Parameter (CAP) and circulating metabolic biomarkers. (**A**) Correlations between CAP and biomarkers at baseline. (**B**) Correlations between CAP and biomarkers after dietary intervention. (**C**) Correlations between the changes (Δ) in CAP and the changes (Δ) in the other biomarkers following dietary intervention. Each cell displays the Spearman correlation coefficient (ρ) and corresponding *p*-value. Red and blue color gradients indicate positive and negative correlations, respectively, with the color intensity reflecting the strength of the association.

**Table 1:** Changes in clinical, anthropometric, BIA and biochemical parameters from baseline to post-VLCKD.

Parameters *	Pre	Post	Δ _After–Before_	*p* ^^^
SBP (mmHg)	130.00 (20.00)	125.00 (10.00)	−5.00 (10.00)	<0.0001
DBP (mmHg)	85.00 (20.00)	80.00 (14.00)	−5.00 (10.00)	<0.0001
Anthropometry				
Weight (Kg)	102.00 (28.00)	92.25 (24.00)	−10.00 (5.00)	<0.0001
BMI (Kg/m^2^)	37.40 (7.12)	33.29 (6.15)	−3.45 (1.65)	<0.0001
Waist (cm)	115.00 (21.50)	105.50 (17.00)	−9.00 (5.00)	<0.0001
Neck (cm)	41.00 (7.00)	38.00 (3.00)	−2.00 (3.00)	0.002
BIA				
RZ (Ω)	447.70 (92.75)	474.00 (118.40)	18.50 (59.05)	0.01
XC (Ω)	50.50 (13.40)	52.10 (10.80)	3.50 (9.80)	0.13
PhA (°)	6.50 (0.90)	6.40 (1.20)	0.00 (0.90)	0.99
BCMI (kg/m^2^)	12.55 (2.60)	11.95 (3.20)	−0.35 (1.40)	0.05
BMR (kcal/die)	1776.00 (288.10)	1696.05 (378.80)	−29.85 (115.60)	0.06
BCM (Kg)	35.40 (9.90)	32.65 (13.10)	−1.05 (4.00)	0.03
FFM (Kg)	61.60 (19.10)	58.30 (18.80)	−3.40 (4.90)	0.0002
FM (Kg)	40.70 (20.77)	34.45 (17.30)	−7.70 (4.80)	<0.0001
TBW (L)	44.00 (14.10)	43.45 (13.40)	−2.70 (3.50)	0.002
ECW (L)	20.65 (5.90)	18.85 (5.30)	−1.00 (2.50)	0.007
Blood				
Glucose (mg/dL)	94.50 (13.00)	91.00 (10.00)	−3.00 (9.00)	0.003
HbA1c (mmol/mol)	37.00 (4.00)	34.00 (4.00)	−3.00 (2.00)	<0.0001
HbA1c (%)	5.50 (0.30)	5.30 (0.30)	−0.30 (0.25)	<0.0001
Creatinine (mg/dL)	0.74 (0.25)	0.80 (0.20)	0.04 (0.07)	0.01
eGFR (mL/min/1.73 m^2^)	90.10 (6.10)	89.00 (12.10)	0.00 (4.00)	0.004
Total Protein (g/dL)	7.00 (0.50)	6.90 (0.50)	−0.10 (0.40)	0.06
Sodium (mmol/L)	140.00 (3.00)	140.00 (2.00)	1.00 (2.00)	0.002
Potassium (mmol/L)	4.36 (0.33)	4.40 (0.35)	0.04 (0.50)	0.75
Calcium (mg/dL)	4.68 (0.18)	4.70 (0.38)	−0.02 (0.34)	0.99
Phosphorus (mg/dL)	3.00 (0.50)	3.30 (0.60)	0.30 (0.60)	0.002
Iron (µg/dL)	79.20 (30.70)	66.90 (29.00)	−12.70 (33.10)	0.004
Cholesterol (mg/dL)	189.00 (34.00)	165.00 (48.00)	−30.00 (34.00)	<0.0001
HDL (mg/dL)	47.25 (16.70)	42.35 (9.20)	−4.75 (8.10)	<0.0001
LDL (mg/dL)	132.20 (30.40)	103.10 (45.80)	−27.20 (29.70)	<0.0001
Triglycerides (mg/dL)	95.00 (52.00)	78.50 (36.50)	−17.50 (50.50)	0.003
TSH (UI/mL)	1.57 (0.79)	1.87 (0.96)	0.15 (0.54)	0.15
FT3 (pg/mL)	3.30 (0.50)	2.9 (0.56)	−0.40 (0.70)	0.002
FT4 (ng/dL)	1.19 (0.18)	1.32 (0.19)	0.12 (0.17)	<0.0001
anti-TPO (UI/mL)	9.90 (3.60)	11.05 (5.48)	0.47 (6.20)	0.05
NT-proBNP (pg/mL)	28.00 (39.00)	41.00 (42.00)	6.50 (32.00)	0.06
Testosterone (ng/dL)	64.90 (329.00)	106.80 (393.35)	12.665 (68.95)	0.003
Prolactin (ng/mL)	8.76 (5.51)	8.09 (6.17)	−0.50 (3.39)	0.63
Vitamin D (ng/mL)	21.70 (11.70)	24.40 (12.20)	2.75 (8.40)	0.11
Insulin (µUI/L)	19.60 (13.20)	10.50 (8.79)	−7.49 (11.71)	<0.0001
HOMA-IR	4.75 (3.16)	2.25 (2.15)	−2.09 (2.63)	<0.0001
PTH (pg/mL)	53.80 (23.30)	48.70 (22.70)	−1.05 (14.30)	0.75
RBC (×10^6^/µL)	5.02 (0.81)	5.08 (0.83)	0.08 (0.25)	0.04
Hemoglobin (g/dL)	14.15 (2.40)	14.70 (2.20)	0.20 (0.60)	0.03
Hematocrit (%)	43.10 (6.25)	44.40 (4.70)	0.50 (1.75)	0.11
MCV (fL)	87.70 (8.55)	87.40 (9.00)	−0.25 (2.00)	0.75
MCH (pg)	28.75 (2.75)	28.85 (2.55)	−0.10 (0.80)	0.63
MCHC (g/dL)	32.65 (1.95)	32.80 (1.80)	0.00 (0.95)	0.99
RDW-CV (%)	13.15 (1.35)	13.20 (1.45)	0.20 (0.75)	0.14
Platelets (×10^3^/µL)	279.00 (64.00)	242.50 (67.50)	−29.0 (41.50)	<0.0001
WBC (×10^3^/µL)	6.67 (2.42)	6.16 (2.19)	−0.60 (1.66)	0.04
Neutrophils (×10^3^/µL)	3.85 (1.43)	3.55 (1.82)	−0.26 (1.32)	0.63
Neutrophils (%)	56.15 (10.00)	56.35 (10.65)	0.70 (6.25)	0.27
Lymphocytes (×10^3^/µL)	2.04 (0.74)	1.82 (0.77)	−0.26 (0.31)	<0.0001
Lymphocytes (%)	33.50 (9.70)	32.20 (9.50)	−0.60 (6.40)	0.87
Monocytes (×10^3^/µL)	0.50 (0.21)	0.45 (0.23)	−0.03 (0.10)	0.20
Monocytes (%)	7.85 (1.75)	8.00 (2.30)	0.10 (1.90)	0.52
Eosinophils (×10^3^/µL)	0.13 (0.11)	0.11 (0.12)	−0.02 (0.07)	0.10
Eosinophils (%)	2.05 (1.45)	2.00 (2.05)	−0.20 (1.30)	0.34
Basophils (×10^3^/µL)	0.04 (0.02)	0.03 (0.02)	−0.01 (0.01)	0.01
Basophils (%)	0.60 (0.25)	0.60 (0.25)	0.00 (0.25)	0.72
Albumin (g/dL)	4.10 (0.40)	4.10 (0.30)	−0.10 (0.20)	0.06
Albumin (%)	59.20 (4.20)	59.72 (4.10)	0.20 (1.50)	0.31
α1 (g/dL)	0.30 (0.10)	0.30 (0.00)	0.00 (0.00)	0.29
α1 (%)	3.80 (0.80)	4.00 (0.60)	0.10 (0.40)	0.30
α2 (g/dL)	0.70 (0.10)	0.60 (0.10)	0.00 (0.10)	0.003
α2 (%)	9.40 (1.50)	9.05 (1.35)	−0.30 (0.60)	0.0003
β1 (g/dL)	0.40 (0.10)	0.40 (0.10)	0.00 (0.00)	0.35
β1 (%)	6.30 (0.60)	6.10 (0.60)	−0.30 (0.50)	0.001
β2 (g/dL)	0.50 (0.00)	0.40 (0.10)	0.00 (0.10)	0.45
β2 (%)	5.70 (0.80)	5.55 (0.95)	−0.10 (0.40)	0.30
γ (g/dL)	1.00 (0.20)	1.00 (0.25)	0.00 (0.00)	0.79
γ (%)	14.70 (2.80)	15.15 (3.20)	0.10 (1.10)	0.32
A/G	1.45 (0.27)	1.48 (0.25)	0.01 (0.09)	0.31

* As Median and Interquartile Range for continuous variables. Abbreviations: PAS, Systolic Blood Pressure; PAD, Diastolic Blood Pressure; BMI, Body Mass Index; RZ, Resistance; XC, Reactance; PhA, Phase Angle; BCMI, Body Cell Mass Index; BMR, Basal Metabolic Rate; BCM, Body Cell Mass; FFM, Fat-Free Mass; FM, Fat Mass; TBW, Total Body Water; ECW, Extracellular Water; HbA1c, Glycated Hemoglobin; eGFR, Estimated Glomerular Filtration Rate; HDL, High-Density Lipoprotein Cholesterol; LDL, Low-Density Lipoprotein Cholesterol; TSH, Thyroid-Stimulating Hormone; FT3, Free Triiodothyronine; FT4, Free Thyroxine; anti-TPO, Anti-Thyroid Peroxidase Antibodies; NT-proBNP, *N*-terminal Pro-B-type Natriuretic Peptide; HOMA, Homeostatic Model Assessment of Insulin Resistance; PTH, Intact Paratyroid Hormone; RBC, Red Blood Cell Count; MCV, Mean Corpuscular Volume; MCH, Mean Corpuscular Hemoglobin; MCHC, Mean Corpuscular Hemoglobin Concentration; RDW-CV, Red Cell Distribution Width—Coefficient of Variation; WBC, White Blood Cell Count. ^^^ Wilcoxon matched-pairs signed-rank test.

**Table 2:** Changes in liver parameters from baseline to post-VLCKD.

Parameters *	Pre	Post	Δ _After–Before_	*p* ^^^
Uric Acid (mg/dL)	5.05 (2.10)	6.10 (1.90)	1.00 (1.60)	0.0001
AST (U/L)	18.50 (9.00)	18.00 (7.00)	−1.00 (6.00)	0.07
ALT (U/L)	25.50 (19.00)	21.00 (17.00)	−3.00 (13.00)	0.11
GGT (U/L)	22.50 (17.00)	15.00 (9.00)	−6.00 (14.00)	<0.0001
CRP (mg/L)	0.35 (0.51)	0.39 (0.37)	−0.04 (0.17)	0.04
Ferritin (ng/mL)	98.30 (221.50)	120.50 (247.45)	4.20 (64.10)	0.87
*Fibroscan*				
CAP (dB/m)	321.00 (62.00)	256.00 (89.00)	−58.00 (59.00)	<0.0001
E (kPa)	5.60 (2.40)	5.60 (4.10)	−0.60 (2.90)	0.0005

* As Median and Interquartile Range for continuous variables. Abbreviations: AST, Aspartate Aminotransferase; ALT, Alanine Aminotransferase; GGT, Gamma-Glutamyl Transferase; CRP, C-Reactive Protein; CAP, Controlled Attenuation Parameter; E, Liver Stiffness. ^^^ Wilcoxon matched-pairs signed-rank test.

**Table 3:** Changes in biomarkers parameters from baseline to post-VLCKD.

Parameters *	Pre	Post	Δ _After–Before_	*p* ^^^
Follistatin (ng/mL)	16.05 (11.46)	15.67 (10.51)	−0.46 (5.25)	0.42
Resistin (pg/mL)	10,530.00 (6537.00)	11,280.00 (9978.00)	70.00 (6611.00)	0.99
SHBG (pmol/L)	32,530.00 (17,740.00)	41,970.00 (30,190.00)	10,652.00 (18,180.00)	<0.0001
Chemerin (ng/mL)	21.35 (13.71)	19.85 (7.65)	−2.24 (8.72)	0.005
PAI-1 (pg/mL)	3241.00 (1365.00)	2953.00 (1442.00)	−359.00 (1109.00)	0.01
Visfatin (ng/mL)	0.94 (0.98)	0.80 (0.98)	−0.15 (0.74)	0.22
FGF-21 (pg/mL)	135.00 (279.85)	103.10 (208.97)	−30.84 (110.84)	0.02
RBP4 (µg/mL)	28.97 (19.86)	22.69 (15.08)	−5.27 (14.85)	0.006
Leptin (ng/mL)	23.29 (21.46)	8.95 (10.19)	−10.29 (11.88)	<0.0001
Adiponectin (ng/mL)	3610.00 (1686.00)	3236.00 (1372.00)	−196.00 (1116.00)	0.10
LPA (µg/mL)	5.55 (1.71)	6.01 (2.75)	0.13 (2.20)	0.79
ATX (ng/mL)	311.80 (128.00)	278.40 (161.00)	−8.20 (69.20)	0.34

* As Median and Interquartile Range for continuous variables. Abbreviations: SHBG, Sex Hormone-Binding Globulin; PAI-1, Plasminogen Activator Inhibitor-1; FGF-21, Fibroblast Growth Factor 21; RBP4, Retinol-Binding Protein 4; LPA, Lysophosphatidic Acid; ATX, Autotaxin. ^^^ Wilcoxon matched-pairs signed-rank test.

## Data Availability

The original data presented in this study are openly available in FigShare at https://doi.org/10.6084/m9.figshare.33393535.

## Supplementary Materials

The following supporting information can be downloaded at: https://www.mdpi.com/article/10.3390/nu18203397/s1, Table S1: Demographic and Lifestyle Characteristics of the Study Population (*n* = 55); Table S2: Proteins assayed and limits of detection; Table S3: Changes in clinical, anthropometric, DEXA and biochemical parameters from baseline to post-VLCKD diet, by sex; Table S4: Changes in liver parameters from baseline to post-VLCKD diet, by sex; Table S5: Changes in biomarkers parameters from baseline to post-VLCKD diet, by sex.
