# Supplementary material for: Short-Term Changes in Metabolic Dysfunction-Associated Steatotic Liver Disease (MASLD) and Related Circulating Biomarkers Following a Very-Low Calorie Ketogenic Diet (VLCKD): A Paired Longitudinal Analysis
Source: Nutrients. 2026 Oct 16;18(20):3397. doi: 10.3390/nu18203397 (PMC13708398; doi:10.3390/nu18203397)
Supplement: Supplementary file 1 [file nutrients-18-03397-s001.zip › Supplementary Materials_RD.pdf]

Supplementary Table S1. Demographic and Lifestyle Characteristics of the Study Population (n=55).

Parameters *	p50 (IQR) or %
Age (yrs)	43.16 (18.32)
Sex (M) (%)	24 (43.64)
Education levels (%)	
No Education	0 (0.00)
Primary School	0 (0.00)
Middle School	7 (17.95)
High School	19 (48.72)
Graduate	11 (28.21)
Post-Graduate	2 (5.13)
Civil Status (%)	
Single	17 (40.48)
Married or Cohabiting	25 (59.52)
Divorced or Separated	0 (0.00)
Widow/er	0 (0.00)
Smoke (%)	
No	21 (50.00)
Ex	7 (16.67)
Yes	14 (33.33)

* As Median and Interquartile Range for continuous variables, and frequency and percentage (%) for categorical.

Supplementary Table S2. Proteins assayed and limits of detection.

Hepatokines	Limit of Detection
Follistatin	500.00 pg/ml
Resistin	3.10 pg/ml
SHBG	1.20 pmol/l
Chemerin	0.50 ng/ml
PAI-1	<30.00 pg/ml
Visfatin	1.10 ng/ml
FGF21	8.00 pg/ml
RBP4	0.034 ng/ml
Leptin	<0.30 pg/ml
Adiponectin	100.00 pg/ml

Supplementary Table S3. Changes in clinical, anthropometric, DEXA and biochemical parameters from baseline to post-VLCKD diet, by sex.

Parameters *	Male (n=24)				Female (n=31)			
	Pre	Post	Δ After-Before	p ^	Pre	Post	Δ After-Before	p ^
SBP (mmHg)	135.00 (10.00)	125.00 (10.00)	-9.00 (5.00)	<0.0001	130.00 (15.00)	125.00 (10.00)	-5.00 (15.00)	<0.0001
DBP (mmHg)	90.00 (15.00)	80.00 (10.00)	-9.00 (5.00)	<0.0001	80.00 (20.00)	80.00 (15.00)	-5.00 (15.00)	<0.0001
Anthropometry								
Weight (Kg)	114.25 (50.75)	102.00 (37.00)	-11.00 (5.00)	<0.0001	98.00 (18.00)	88.50 (16.00)	-8.50 (5.50)	<0.0001
BMI (Kg/m²)	37.64 (12.84)	33.00 (13.01)	-3.62 (1.70)	<0.0001	36.88 (4.15)	33.56 (5.41)	-3.16 (2.05)	<0.0001
Waist (cm)	123.00 (18.00)	110.50 (16.50)	-10.50 (4.00)	<0.0001	110.00 (10.00)	101.00 (14.00)	-8.00 (4.00)	<0.0001
Neck (cm)	45.00 (9.00)	39.00 (1.00)	-2.00 (2.00)	0.06	38.00 (3.00)	36.00 (2.00)	-1.00 (3.00)	0.06
BIA								
RZ (Ω)	403.00 (67.00)	409.95 (100.20)	20.00 (23.30)	0.05	473.00 (66.20)	510.00 (114.55)	16.00 (72.60)	0.17
XC (Ω)	48.80 (10.80)	52.00 (13.00)	3.50 (8.10)	0.33	53.00 (15.00)	53.00 (12.50)	3.50 (12.80)	0.36
PhA (°)	6.70 (0.70)	6.65 (1.50)	0.10 (0.90)	0.80	6.10 (1.40)	6.10 (0.85)	-0.05 (0.70)	0.99
BCMI (kg/m²)	14.00 (1.90)	14.20 (3.90)	-0.50 (2.10)	0.63	11.60 (2.20)	11.00 (1.75)	-0.30 (0.70)	0.03
BMR (kcal/die)	1940.40 (195.80)	1950.55 (314.70)	-42.60 (175.70)	0.63	1652.30 (156.20)	1563.00 (159.20)	-25.80 (58.90)	0.05
BCM (Kg)	41.00 (6.80)	41.40 (10.80)	-1.50 (6.10)	0.63	31.10 (5.40)	28.05 (5.50)	-0.90 (2.00)	0.02
FFM (Kg)	75.10 (10.50)	69.95 (10.10)	-3.70 (5.50)	0.02	56.85 (5.30)	52.10 (6.30)	-3.20 (3.40)	0.007
FM (Kg)	38.10 (12.80)	32.00 (28.30)	-7.70 (6.30)	0.0003	44.55 (15.70)	38.30 (13.45)	-7.10 (4.30)	<0.0001
TBW (L)	54.90 (8.00)	51.25 (8.00)	-3.60 (1.90)	0.01	41.05 (6.40)	38.35 (4.90)	-1.55 (3.60)	0.10
ECW (L)	22.60 (3.70)	22.35 (4.50)	-1.10 (2.70)	0.01	19.00 (3.40)	17.25 (3.40)	-0.45 (2.20)	0.30
Blood								
Glucose (mg/dL)	97.00 (16.50)	93.50 (10.50)	-4.00 (9.50)	0.03	93.50 (12.00)	90.00 (11.00)	-3.00 (10.00)	0.08
HbA1c (mmol/mol)	37.00 (3.50)	34.00 (3.00)	-3.00 (2.00)	<0.0001	36.50 (4.00)	34.00 (4.00)	-3.00 (4.00)	0.0002

(continue)

Parameters *	Male (n=24)				Female (n=31)			
	Pre	Post	Δ After-Before	p ^	Pre	Post	Δ After-Before	p ^
HbA1c (%)	5.50 (0.35)	5.30 (0.30)	-0.30 (0.20)	0.0001	5.50 (0.35)	5.30 (0.30)	-0.25 (0.30)	0.0001
Creatinine (mg/dL)	0.93 (0.18)	0.90 (0.20)	0.04 (0.10)	0.24	0.67 (0.08)	0.72 (0.11)	0.03 (0.07)	0.03
eGFR (mL/min/1,73 m²)	89.50 (7.10)	89.05 (13.10)	0.00 (4.50)	0.15	90.10 (6.10)	89.00 (9.10)	0.00 (3.10)	0.02
Total Protein (g/dL)	7.00 (0.50)	6.95 (0.55)	-0.10 (0.40)	0.33	7.00 (0.60)	6.90 (0.60)	-0.20 (0.40)	0.17
Sodium (mmol/L)	140.00 (2.50)	140.50 (1.00)	1.00 (2.50)	0.14	140.00 (2.00)	140.00 (2.00)	1.00 (2.00)	0.006
Potassium (mmol/L)	4.40 (0.34)	4.37 (0.31)	0.07 (0.51)	0.26	4.24 (0.25)	4.42 (0.55)	-0.02 (0.52)	0.66
Calcium (mg/dL)	4.74 (0.20)	4.73 (0.45)	0.03 (0.30)	0.82	4.64 (0.16)	4.62 (0.36)	-0.04 (0.34)	0.66
Phosphorus (mg/dL)	2.90 (0.45)	3.30 (0.70)	0.30 (0.60)	0.04	3.05 (0.30)	3.30 (0.50)	0.20 (0.60)	0.04
Iron (μg/dL)	83.85 (35.00)	65.60 (25.30)	-19.70 (30.70)	0.003	71.60 (41.30)	68.80 (33.90)	-5.40 (37.00)	0.38
Cholesterol (mg/dL)	204.00 (26.50)	174.50 (44.00)	-22.00 (28.00)	<0.0001	177.50 (27.00)	151.00 (56.00)	-37.00 (41.00)	0.003
HDL (mg/dL)	44.70 (10.55)	41.15 (8.35)	-3.55 (6.05)	0.003	54.25 (16.90)	43.30 (9.65)	-9.20 (9.30)	0.0004
LDL (mg/dL)	142.35 (17.30)	115.85 (44.75)	-30.50 (32.20)	0.0004	120.10 (23.20)	92.50 (39.10)	-27.20 (27.70)	0.003
Triglycerides (mg/dL)	107.50 (108.00)	84.50 (58.00)	-22.50 (56.00)	0.04	89.00 (26.00)	73.50 (19.00)	-11.00 (36.50)	0.06
TSH (μIU/mL)	1.43 (0.70)	1.41 (0.86)	0.15 (0.43)	0.50	1.80 (0.72)	2.01 (0.99)	0.14 (0.87)	0.26
FT3 (pg/mL)	3.46 (0.61)	2.84 (0.46)	-0.47 (0.51)	0.0004	3.13 (0.55)	3.00 (0.72)	-0.24 (0.79)	0.50
FT4 (ng/dL)	1.23 (0.17)	1.36 (0.12)	0.11 (0.13)	0.0007	1.11 (0.23)	1.25 (0.18)	0.14 (0.16)	0.0004
anti-TPO (IU/mL)	10.55 (3.30)	9.69 (2.85)	0.00 (2.85)	0.99	9.19 (4.30)	12.35 (6.65)	2.05 (6.45)	0.003
NT-proBNP (pg/mL)	18.50 (32.50)	32.50 (32.50)	5.50 (17.50)	0.26	32.00 (31.00)	48.00 (45.00)	12.50 (35.00)	0.21
Testosterone (ng/dL)	354.00 (130.50)	415.50 (212.00)	68.50 (129.50)	<0.0001	19.00 (18.60)	22.15 (22.20)	2.70 (12.10)	0.99
Prolactin (ng/mL)	8.54 (3.96)	7.91 (5.45)	-0.50 (2.64)	0.50	9.48 (7.96)	9.95 (9.38)	-0.38 (5.26)	0.99
Vitamin D (ng/mL)	22.15 (11.55)	22.85 (11.40)	1.15 (10.70)	0.50	21.10 (11.40)	24.90 (13.20)	3.05 (8.20)	0.17
Insulin (μUI/L)	20.45 (12.65)	9.92 (7.97)	-6.55 (9.57)	<0.0001	18.90 (13.50)	10.60 (9.28)	-10.69 (15.08)	0.003
HOMA-IR	5.05 (2.97)	2.26 (2.12)	-1.71 (2.70)	<0.0001	4.15 (3.33)	2.25 (1.95)	-2.68 (3.06)	0.003

(continue)

Parameters *	Male (n=24)				Female (n=31)			
	Pre	Post	Δ After-Before	p ^	Pre	Post	Δ After-Before	p ^
PTH (pg/mL)	48.25 (19.90)	45.85 (20.60)	1.20 (11.00)	0.82	62.15 (23.00)	54.25 (28.55)	-5.35 (18.95)	0.36
RBC (×10⁶/μL)	5.22 (0.44)	5.28 (0.61)	0.01 (0.24)	0.82	4.66 (0.98)	4.73 (0.99)	0.09 (0.20)	0.01
Hemoglobin (g/dL)	15.50 (1.15)	15.50 (1.10)	0.15 (0.80)	0.65	13.15 (1.65)	13.30 (1.00)	0.30 (0.55)	0.02
Hematocrit (%)	46.55 (3.65)	45.90 (3.25)	-0.05 (2.35)	0.99	40.40 (3.70)	41.20 (2.95)	0.95 (2.00)	0.01
MCV (fL)	88.15 (6.25)	87.60 (4.65)	-0.20 (1.55)	0.99	86.10 (10.85)	86.50 (10.60)	-0.25 (2.15)	0.82
MCH (pg)	29.25 (1.65)	29.50 (1.85)	-0.20 (1.10)	0.82	27.70 (4.20)	27.95 (3.55)	-0.05 (0.65)	0.81
MCHC (g/dL)	33.20 (1.65)	33.35 (1.85)	0.20 (0.85)	0.50	32.10 (1.75)	32.00 (1.95)	-0.10 (0.90)	0.48
RDW-CV (%)	12.80 (0.75)	13.20 (0.75)	0.20 (0.65)	0.36	13.40 (2.30)	13.35 (2.90)	0.30 (1.05)	0.36
Platelets (×10³/μL)	258.00 (42.50)	232.00 (39.50)	-27.00 (36.50)	0.003	297.00 (70.50)	251.50 (87.50)	-37.50 (59.50)	0.003
WBC (×10³/μL)	6.82 (2.01)	6.14 (1.52)	-0.61 (1.58)	0.11	6.47 (2.56)	6.32 (2.81)	-0.60 (1.99)	0.26
Neutrophils (×10³/μL)	3.96 (1.05)	3.54 (1.81)	-0.18 (1.16)	0.99	3.48 (1.80)	3.55 (1.83)	-0.26 (1.31)	0.50
Neutrophils (%)	57.10 (7.65)	56.10 (10.10)	1.50 (4.95)	0.11	55.30 (9.35)	56.65 (10.70)	0.00 (9.05)	0.99
Lymphocytes (×10³/μL)	2.09 (0.77)	1.60 (0.77)	-0.30 (0.28)	0.0004	2.04 (0.62)	1.86 (0.82)	-0.22 (0.49)	0.04
Lymphocytes (%)	29.90 (7.35)	32.10 (8.40)	-1.35 (5.70)	0.50	34.05 (8.95)	32.20 (10.55)	0.50 (6.80)	0.82
Monocytes (×10³/μL)	0.54 (0.22)	0.44 (0.13)	-0.04 (0.13)	0.17	0.47 (0.22)	0.49 (0.26)	-0.01 (0.11)	0.82
Monocytes (%)	7.95 (2.25)	8.05 (2.55)	-0.05 (2.20)	0.99	7.65 (1.50)	7.95 (2.15)	0.40 (1.95)	0.26
Eosinophils (×10³/μL)	0.14 (0.07)	0.13 (0.09)	-0.01 (0.08)	0.26	0.09 (0.11)	0.09 (0.14)	-0.03 (0.06)	0.33
Eosinophils (%)	2.20 (0.85)	2.50 (1.45)	-0.20 (1.45)	0.82	1.90 (2.10)	1.55 (2.40)	-0.30 (1.20)	0.36
Basophils (×10³/μL)	0.04 (0.02)	0.03 (0.01)	-0.01 (0.01)	0.03	0.04 (0.02)	0.04 (0.03)	0.00 (0.01)	0.27
Basophils (%)	0.60 (0.25)	0.55 (0.20)	0.00 (0.25)	0.42	0.60 (0.30)	0.60 (0.25)	0.00 (0.25)	0.99
Albumin (g/dL)	4.30 (0.45)	4.20 (0.40)	-0.10 (0.25)	0.21	4.00 (0.30)	4.00 (0.25)	0.00 (0.20)	0.27
Albumin (%)	62.20 (4.95)	61.75 (3.80)	0.00 (1.60)	0.99	58.40 (2.50)	58.80 (3.35)	0.20 (1.50)	0.14

(continue)

Parameters *	Male (n=24)				Female (n=31)			
	Pre	Post	Δ After-Before	p ^	Pre	Post	Δ After-Before	p ^
α1 (g/dL)	0.20 (0.10)	0.30 (0.10)	0.00 (0.05)	0.06	0.30 (0.00)	0.30 (0.00)	0.30 (0.00)	0.99
α1 (%)	3.55 (0.65)	3.75 (0.45)	0.25 (0.50)	0.14	4.10 (0.80)	4.10 (0.45)	0.00 (0.50)	0.99
α2 (g/dL)	0.60 (0.10)	0.60 (0.00)	0.00 (0.10)	0.29	0.70 (0.00)	0.65 (0.10)	-0.10 (0.10)	0.006
α2 (%)	9.00 (1.35)	8.90 (1.10)	-0.20 (0.60)	0.11	10.00 (1.30)	9.45 (1.60)	-0.60 (0.70)	0.0007
β1 (g/dL)	0.40 (0.10)	0.40 (0.05)	0.00 (0.00)	0.69	0.40 (0.10)	0.40 (0.10)	0.0 (0.00)	0.62
β1 (%)	6.20 (0.60)	6.10 (0.60)	-0.20 (0.40)	0.007	6.30 (0.70)	6.30 (0.70)	-0.30 (0.70)	0.10
β2 (g/dL)	0.40 (0.05)	0.40 (0.00)	0.00 (0.00)	0.99	0.40 (0.00)	0.40 (0.10)	0.00 (0.10)	0.29
β2 (%)	5.75 (1.00)	5.65 (0.85)	-0.05 (0.50)	0.81	5.50 (0.70)	5.35 (1.10)	-0.10 (0.40)	0.30
γ (g/dL)	0.95 (0.15)	1.00 (0.25)	0.00 (0.05)	0.45	1.10 (0.20)	1.10 (0.25)	0.00 (0.00)	0.99
γ (%)	13.85 (2.00)	14.25 (2.70)	-0.05 (1.20)	0.99	15.70 (2.30)	16.20 (2.90)	0.50 (0.90)	0.10
A/G	1.65 (0.33)	1.61 (0.26)	0.00 (0.10)	0.99	1.40 (0.15)	1.43 (0.19)	0.01 (0.09)	0.14

* As Median and Interquartile Range for continuous variables.

Abbreviations: PAS, Systolic Blood Pressure; PAD, Diastolic Blood Pressure; BMI, Body Mass Index; RZ, Resistance; XC, Reactance; PhA, Phase Angle; BCMI, Body Cell Mass Index; BMR, Basal Metabolic Rate; BCM, Body Cell Mass; FFM, Fat-Free Mass; FM, Fat Mass; TBW, Total Body Water; ECW, Extracellular Water; HbA1c, Glycated Hemoglobin; eGFR, Estimated Glomerular Filtration Rate; HDL, High-Density Lipoprotein Cholesterol; LDL, Low-Density Lipoprotein Cholesterol; TSH, Thyroid-Stimulating Hormone; FT3, Free Triiodothyronine; FT4, Free Thyroxine; anti-TPO, Anti-Thyroid Peroxidase Antibodies; NT-proBNP, N-terminal Pro-B-type Natriuretic Peptide; HOMA, Homeostatic Model Assessment of Insulin Resistance; PTH, Intact Paratyroid Hormone; RBC, Red Blood Cell Count; MCV, Mean Corpuscular Volume; MCH, Mean Corpuscular Hemoglobin; MCHC, Mean Corpuscular Hemoglobin Concentration; RDW-CV, Red Cell Distribution Width – Coefficient of Variation; WBC, White Blood Cell Count.

^ Wilcoxon matched-pairs signed-rank test.

Supplementary Table S4. Changes in liver parameters from baseline to post-VLCKD diet, by sex.

Parameters *	Male (n=24)				Female (n=31)			
	Pre	Post	Δ After-Before	p ^	Pre	Post	Δ After-Before	p ^
Uric Acid	6.35 (1.70)	7.05 (2.10)	0.80 (1.65	0.11	4.70 (0.90)	5.60 (1.40)	1.10 (1.60)	0.0002
AST	20.00 (11.00)	18.50 (12.50)	-3.00 (8.00)	0.02	17.50 (7.00)	17.00 (5.00)	0.00 (4.00)	0.99
ALT	29.00 (17.00)	24.00 (19.00)	-6.50 (17.50)	0.11	19.00 (18.00)	20.00 (8.00)	-2.00 (6.00)	0.65
GGT	30.50 (28.50)	19.00 (11.00)	-11.00 (22.50)	<0.0001	18.50 (14.00)	14.00 (7.00)	-5.00 (8.00)	<0.0001
CRP	0.27 (0.31)	0.23 (0.30)	-0.03 (0.16)	0.82	0.57 (0.40)	0.45 (0.43)	-0.10 (0.20)	0.01
Ferritin	284.00 (404.35)	300.50 (249.50)	-10.65 (107.30)	0.26	45.10 (70.30)	68.00 (114.10)	7.10 (43.15)	0.11
Fibroscan								
CAP	333.00 (63.00)	282.00 (100.50)	-59.00 (63.00)	<0.0001	301.00 (62.00)	239.00 (74.00)	-58.00 (54.00)	<0.0001
E	5.80 (2.10)	6.40 (4.35)	-1.35 (4.20)	0.21	5.10 (2.70)	5.50 (2.60)	-0.50 (1.40)	0.0009

* As Median and Interquartile Range for continuous variables.

Abbreviations: AST, Aspartate Aminotransferase; ALT, Alanine Aminotransferase; GGT, Gamma-Glutamyl Transferase; CRP, C-Reactive Protein; CAP, Controlled Attenuation Parameter; E, Liver Stiffness.

^ Wilcoxon matched-pairs signed-rank test.

Table S5. Changes in biomarkers parameters from baseline to post-VLCKD diet, by sex.

Parameters *	Male (n=24)				Female (n=31)			
	Pre	Post	Δ After-Before	p ^	Pre	Post	Δ After-Before	p ^
Follistatin	15.43 (9.35)	15.41 (-0.97)	-0.97 (4.64)	0.15	16.67 (12.23)	15.78 (13.24)	0.26 (5.72)	0.99
Resistin	10685.00 (7807.00)	[illegible]1290.00 (10419.50)	847.00 (6567.50)	0.54	10500.00 (6537.00)	10220.00 (10080.00)	-262.00 (6140.00)	0.72
SHBG	29270.00 (16340.00)	39640.00 (24735.00)	10481.00 (13875.00)	0.007	33170.00 (32900.00)	44110.00 (52030.00)	13490.00 (26210.00)	0.0002
Chemerin	20.41 (10.44)	18.12 (5.59)	-4.02 (7.04)	0.007	22.80 (77.95)	20.75 (78.69)	-2.22 (9.49)	0.26
PAI-1	3373.50 (1222.00)	2893.50 (1080.00)	-516.50 (1227.50)	0.15	3193.00 (1826.00)	3008.00 (2133.00)	-260.00 (984.00)	0.07
Visfatin	0.89 (0.54)	0.71 (0.25)	-0.02 (0.69)	0.99	1.06 (2.72)	0.94 (2.41)	-0.18 (0.81)	0.10
FGF-21	104.90 (160.01)	94.52 (84.64)	-28.07 (109.64)	0.15	149.00 (450.57)	157.00 (421.70)	-30.84 (111.15)	0.10
RBP4	34.72 (14.68)	27.45 (21.41)	-7.37 (18.21)	0.15	21.59 (14.13)	18.94 (11.12)	-3.99 (10.77)	0.03
Leptina	15.27 (10.99)	6.17 (4.36)	-7.56 (7.30)	<0.0001	30.02 (16.91)	13.56 (9.77)	-14.77 (10.34)	<0.0001
Adiponectin	3054.00 (1681.60)	2668.00 (1487.00)	-166.20 (1285.20)	0.54	4036.00 (1840.00)	3732.00 (1156.00)	-236.00 (1174.00)	0.15
LPA	5.72 (1.36)	6.01 (2.15)	-0.11 (2.79)	0.54	5.52 (2.47)	6.01 (2.84)	0.46 (1.89)	0.28
ATX	272.20 (83.30)	251.40 (67.00)	-6.50 (51.68)	0.84	356.80 (103.80)	324.00 (152.40)	-21.80 (90.40)	0.36

* As Median and Interquartile Range for continuous variables.

Abbreviations: SHBG, Sex Hormone-Binding Globulin; PAI-1, Plasminogen Activator Inhibitor-1; FGF-21, Fibroblast Growth Factor 21; RBP4, Retinol-Binding Protein 4; LPA, Lysophosphatidic Acid; ATX, Autotaxin.

^ Wilcoxon matched-pairs signed-rank test.
